# The Effectiveness of Emergency Obstetric Referral Interventions in Developing Country Settings: A Systematic Review

**DOI:** 10.1371/journal.pmed.1001264

**Published:** 2012-07-10

**Authors:** Julia Hussein, Lovney Kanguru, Margaret Astin, Stephen Munjanja

**Affiliations:** 1 Immpact, University of Aberdeen, Aberdeen, Scotland; 2 University of Bristol, Bristol, England; 3 University of Zimbabwe, Harare, Zimbabwe; The Johns Hopkins Bloomberg School of Public Health, United States of America

## Abstract

**Background:**

Pregnancy complications can be unpredictable and many women in developing countries cannot access health facilities where life-saving care is available. This study assesses the effects of referral interventions that enable pregnant women to reach health facilities during an emergency, after the decision to seek care is made.

**Methods and findings:**

Selected bibliographic databases were searched with no date or language restrictions. Randomised controlled trials and quasi experimental study designs with a comparison group were included. Outcomes of interest included maternal and neonatal mortality and other intermediate measures such as service utilisation. Two reviewers independently selected, appraised, and extracted articles using predefined fields. Forest plots, tables, and qualitative summaries of study quality, size, and direction of effect were used for analysis.

Nineteen studies were included. In South Asian settings, four studies of organisational interventions in communities that generated funds for transport reduced neonatal deaths, with the largest effect seen in India (odds ratio 0·48 95% CI 0·34–0·68). Three quasi experimental studies from sub-Saharan Africa reported reductions in stillbirths with maternity waiting home interventions, with one statistically significant result (OR 0.56 95% CI 0.32–0.96). Effects of interventions on maternal mortality were unclear. Referral interventions usually improved utilisation of health services but the opposite effect was also documented. The effects of multiple interventions in the studies could not be disentangled. Explanatory mechanisms through which the interventions worked could not be ascertained.

**Conclusions:**

Community mobilisation interventions may reduce neonatal mortality but the contribution of referral components cannot be ascertained. The reduction in stillbirth rates resulting from maternity waiting homes needs further study. Referral interventions can have unexpected adverse effects. To inform the implementation of effective referral interventions, improved monitoring and evaluation practices are necessary, along with studies that develop better understanding of how interventions work.

*Please see later in the article for the Editors' Summary*

## Abstract 2

In a systematic review of the literature, Julia Hussein and colleagues seek to determine the effect of referral interventions that enable emergency access to health facilities for pregnant women living in developing countries.

## Editors' Summary

**Background:**

Every year, about 350,000 women die from pregnancy- or childbirth-related complications. Almost all of these “maternal” deaths occur in developing countries. In sub-Saharan Africa, for example, the maternal mortality ratio (MMR, the number of maternal deaths per 100,000 live births) is 500 and a woman's life-time risk of dying from complications of pregnancy or childbirth is 1 in 39. By contrast, the MMR in industrialized countries is 12 and women have a life-time risk of maternal death of 1 in 4,700. Most maternal deaths are caused by hemorrhage (severe bleeding after childbirth), post-delivery infections, obstructed (difficult) labor, and blood pressure disorders during pregnancy, all of which are preventable or treatable conditions. Unfortunately, it is hard to predict which women will develop pregnancy complications, many complications rapidly become life-threatening and, in developing countries, women often deliver at home, far from emergency obstetric services; obstetrics deals with the care of women and their children during pregnancy, childbirth, and the postnatal period.

**Why Was This Study Done?:**

It should be possible to reduce maternal deaths (and the deaths of babies during pregnancy, childbirth, and early life) in developing countries by ensuring that pregnant women are referred to emergency obstetric services quickly when the need arises. Unfortunately, in such countries referral to emergency obstetric care is beset with problems such as difficult geographical terrain, transport costs, lack of vehicles, and suboptimal location and distribution of health care facilities. In this systematic review (a study that uses predefined criteria to identify all the research on a given topic), the researchers assess the effectiveness of interventions designed to reduce the “phase II delay” in referral to emergency obstetric care in developing countries—the time it takes a woman to reach an appropriate health care facility once a problem has been recognized and the decision has been taken to seek care. Delays in diagnosis and the decision to seek care are phase I delays in referral, whereas delays in receiving care once a women reaches a health care facility are phase III delays.

**What Did the Researchers Do and Find?:**

The researchers identified 19 published studies that described 14 interventions designed to overcome phase II delays in emergency obstetric referral and that met their criteria for inclusion in their systematic review. About half of the interventions were organizational. That is, they were designed to overcome barriers to referral such as costs. Most of the remaining interventions were structural. That is, they involved the provision of, for example, ambulances and maternity waiting homes—placed close to a health care facility where women can stay during late pregnancy. Although seven studies provided data on maternal mortality, none showed a sustained, statistically significant reduction (a reduction unlikely to have occurred by chance) in maternal deaths. Four studies in South Asia in which communities generated funds for transport reduced neonatal deaths (deaths of babies soon after birth), but the only statistically significant effect of this community mobilization intervention was seen in India where neonatal deaths were halved. Three studies from sub-Saharan Africa reported that the introduction of maternity waiting homes reduced stillbirths but this reduction was only significant in one study. Finally, although referral interventions generally improved the utilization of health services, in one study the provision of bicycle ambulances to take women to the hospital reduced the proportion of women delivering in health facilities, probably because women felt that bicycle ambulances drew unwanted attention to them during labor and so preferred to stay at home.

**What Do These Findings Mean?:**

These findings suggest that community mobilization interventions may reduce neonatal mortality and that maternity waiting rooms may reduce stillbirths. Importantly, they also highlight how referral interventions can have unexpected adverse effects. However, because the studies included in this systematic review included multiple interventions designed to reduce delays at several stages of the referral process, it is not possible to disentangle the contribution of each component of the intervention. Moreover, it is impossible at present to determine why (or even if) any of the interventions reduced maternal mortality. Thus, the researchers conclude, improved monitoring of interventions and better evaluation of outcomes is essential to inform the implementation of effective referral interventions, and more studies are needed to improve understanding of how referral interventions work.

**Additional Information:**

Please access these Web sites via the online version of this summary at http://dx.doi.org/10.1371/journal.pmed.1001264.

- The United Nations Children's Fund (UNICEF) provides information on maternal mortality, including the WHO/UNICEF./UNFPA/World Bank 2008 country estimates of maternal mortality
- The World Health Organization provides information on maternal health, including information about Millennium Development Goal 5, which aims to reduce maternal mortality (in several languages); the Millennium Development Goals, which were agreed by world leaders in 2000, are designed to eradicate extreme poverty worldwide by 2015
- Immpact is a global research initiative for the evaluation of safe motherhood intervention strategies
- Veil of Tears contains personal stories from Afghanistan about loss in childbirth; the non-governmental health development organization AMREF provides personal stories about maternal health in Africa
- Maternal Death: The Avoidable Crisis is a briefing paper published by Médecins Sans Frontières (MSF) in March 2012

## Introduction

The importance of referral in an obstetric emergency is related to the unpredictability of pregnancy complications and their potential to progress rapidly to become severe and life threatening. For example, a serious haemorrhage can lead to death of a woman and the unborn fetus within minutes or hours [1],[2]. In the poorest countries, two-thirds of women deliver at home, far from emergency services or without access to a health professional [3]. Maternal and neonatal deaths could therefore be prevented if functional referral systems were in place to allow pregnant women to reach appropriate health services when complications occur. A recent systematic review of maternal health initiatives indicated that the most successful programmes included the establishment of referral systems as a component [4].

The three delays model provides a conceptual framework of the factors influencing the timely arrival to appropriate care in obstetric emergencies [5]. The “three delays” are (I) delays in the recognition of the problem and the decision to seek care in the household, (II) delays in reaching the appropriate facility, and (III) delays in the care received once the woman reaches the facility. The delays of interest in this review are the phase II delays—those experienced after the decision to seek care is made, and before obtaining adequate care.

**Figure 1:**
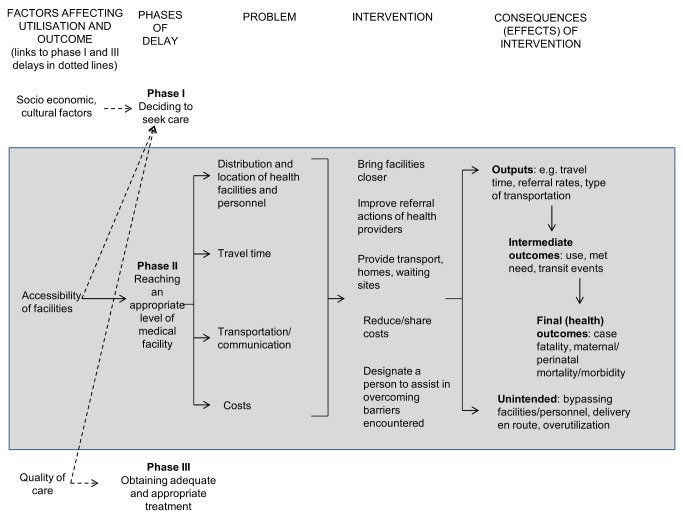
Conceptual framework for the review.

Figure 1 depicts the conceptual framework for this review, which was originally proposed by Thaddeus & Maine [5]. The reasons that phase II delays occur have been well documented and include difficult geographical terrain, costs of transport, lack of phones and vehicles, suboptimal distribution and location of health facilities, and poor decision making of health professionals [5],[6], so interventions usually address these barriers. Although the problems identified in the diagram are presented as distinct from each other, it is likely that they are interlinked, so one intervention might affect more than one problem area or lead to several consequences. For example, if a health facility was placed nearer to women's homes to improve the distribution of services, it is possible that access to a phone or specialised emergency vehicle might also be improved. The new health facility may lead to positive consequences (such as decreased travel time or increased utilisation of the service, which may result in decreased maternal morbidity and mortality), but may also have negative or unintended effects (for example, if the health provider in the facility is over worked and does not effectively carry out triage of cases, s/he may cause delays in referring the most urgent cases).

A large number of interventions to overcome phase II delays are being implemented within maternal mortality reduction programmes. Many report improvements, especially in terms of health service indicators such as referral rates and utilization. Reviews of the literature have so far not attempted to systematically collate and appraise the reported effects. This systematic review aims to assess the effects of emergency obstetric referral interventions that overcome phase II delays, enabling pregnant women to reach health facilities in developing countries.

## Methods

PRISMA guidelines (Text S1) and a study protocol (Text S2) were used as a basis for the overall study approach.

### Inclusion and Exclusion Criteria

Studies of referral systems for emergency maternity care from published and grey literature were included if they were randomised controlled trials (RCTs) or other quasi experimental study designs—i.e., studies lacking randomisation such as controlled before-after (CBA) studies and interrupted time series (ITS)—provided comparison groups were available. Participants were pregnant and post partum women with an obstetric complication, referred as an emergency from the community or from a primary care centre to a higher level comprehensive emergency obstetric care facility. Interventions included aimed to overcome delays in reaching the appropriate facility (phase II delay), which improved emergency referrals antenatally, during labour, or up to 42 d after delivery. Examples of interventions of interest were technologies such as telephones, radios, and vehicles for transport; financing and incentive schemes; guidelines and protocols to help health professionals make decisions on referrals; reorganisation of care systems (such as introducing intermediate services like maternity waiting homes or by linking different referral modalities); and mobilisation of community members (including traditional birth attendants) to actively participate in referral activities (such as accompany the women or drive vehicles). Interventions that addressed phase II delays, combined with those that improved phase I and III delays, were included. Studies from low- and middle-income developing countries as classified by the World Bank [7] were eligible.

Studies with no comparison group, and interventions that referred the newborn, women without maternity related conditions, non-emergency cases, and between hospitals were excluded. Interventions that only improved phase I and III delays were omitted. Studies in refugee, war zone, and mass casualty settings were not included as these special settings were likely to confound the effect of obstetric referral. Interventions to change traditional birth attendants' (and other lay carers of pregnant women) decision making for referral were not included (unless there was active engagement of the carer in enabling transfer to a facility), as we considered these to mainly affect decision making. Isolated interventions to introduce first-aid practices to stabilise or treat the woman during referral (such as training community health workers in special procedures) were excluded as these procedures do not affect the delay in reaching a facility but enable improvement of the woman's condition while awaiting transfer. First aid is, however, normal practice during referral and if it was provided as part of an intervention to reduce phase II delays, the study would be included.

The outcomes of interest were guided by the conceptual framework (Figure 1). These were: maternal and neonatal mortality comprising stillbirths, live births, and case fatalities; intermediate outcomes such as utilisation levels and care for maternal complications (expressed as the “met need for obstetric care,” which is the proportion of complications seen to expected); and outputs or processes such as travel time, referral rates, type of transport or communication, costs (payments for transport, health facility fees, and loss of income), women's knowledge of pregnancy or post partum complications, and satisfaction with the intervention.

### Search Strategy

The electronic search strategy was based on terms related to referral, transport, or transfer, in obstetric emergencies and conducted between July and November 2010. The full search strategy is available as Text S3. It was run in MEDLINE, then adapted for the following databases: EMBASE, CAB abstracts, Cochrane Central Register of Controlled Trials (CENTRAL), CINAHL, and Effective Pregnancy and Organisation of Care (EPOC) by selecting appropriate MeSH and/or keywords from their respective thesauri with no date or language restrictions. The search was subsequently extended to LILACS and the African Index Medicus. Electronic search citations were downloaded using Reference Manager 12. Reference lists from retrieved papers were screened. Published and grey literature was included at this point. Authors were contacted to ask for additional information if required.

### Data Extraction, Quality Assessment, and Analysis

Appraisal of titles, abstracts, and full text articles was conducted independently by two reviewers on the basis of the inclusion and exclusion criteria. Disagreements were resolved by discussion among all members of the review team.

Quality assessment was guided by Effective Public Health Practice Project criteria [8], summarised in Table 1. Two reviewers independently assigned quality scores and compared judgements. Uncertainties were resolved through arbitration with one other reviewer.

**Table 1:** Quality assessment summary table.

Author Year	Selection Bias	Study Design	Confounder	Blinding	Data Collection Methods	Withdrawal/Dropouts	Integrity of intervention^a^
Alisjahbana 1995	Moderate	Moderate	Weak	Weak	Moderate	Strong	Moderate
Azad 2010	Strong	Strong	Strong	Weak	Strong	Strong	Strong
Bailey 2002	Strong	Weak	Weak	Weak	Weak	Strong	Moderate
Bhutta 2008	Strong	Strong	Weak	Moderate	Strong	Weak	Moderate
Brazier 2009/FCI 2007	Moderate	Moderate	Weak	Weak	Moderate	Weak	Moderate
Chandramohan 1995	Moderate	Moderate	Moderate	Weak	Weak	Weak	Moderate
Chandramohan 1994	Moderate	Moderate	Weak	Weak	Weak	Strong	Moderate
Fauveau 1991	Moderate	Weak	Strong	Weak	Moderate	Weak	Strong
Hossain 2006/Barbey 2001	Moderate	Moderate	Weak	Weak	Moderate	Weak	Strong
Kumar 2008	Strong	Strong	Moderate	Weak	Strong	Strong	Strong
Lonkhuijzen 2003	Weak	Moderate	Weak	Weak	Weak	Strong	Moderate
Lungu 2001	Moderate	Moderate	Strong	Weak	Weak	Strong	Moderate
Maine 1996	Moderate	Weak	Weak	Weak	Weak	Weak	Strong
Manandhar 2004	Strong	Strong	Strong	Weak	Strong	Strong	Moderate
Millard 1991	Moderate	Moderate	Weak	Weak	Weak	Weak	Moderate
Ronsmans 1997	Not applicable	Moderate	Moderate	Weak	Strong	Weak	Strong
Tumwine 1996	Weak	Moderate	Weak	Weak	Weak	Weak	Moderate

a Defined as any unintended intervention or inconsistencies between control and intervention arms. Low (<50% comparable between arms), moderate (at least 50% comparable between arms), and strong (>80% comparable between arms).

Synthesis of the studies involved categorising interventions by their characteristics, narrating and summarising study quality, size and direction of effect, and presenting data visually as odds ratios, forest plots, and tables. Data extraction and analyses was done with Review Manager Version 5. In our analysis, the effect estimates of cluster RCTs were corrected to account for this design using inverse variance. Denominators used were based on the total numbers of pregnant women, live births, or total births (in the case of stillbirths) in the catchment areas of the respective studies.

## Results

**Figure 2:**
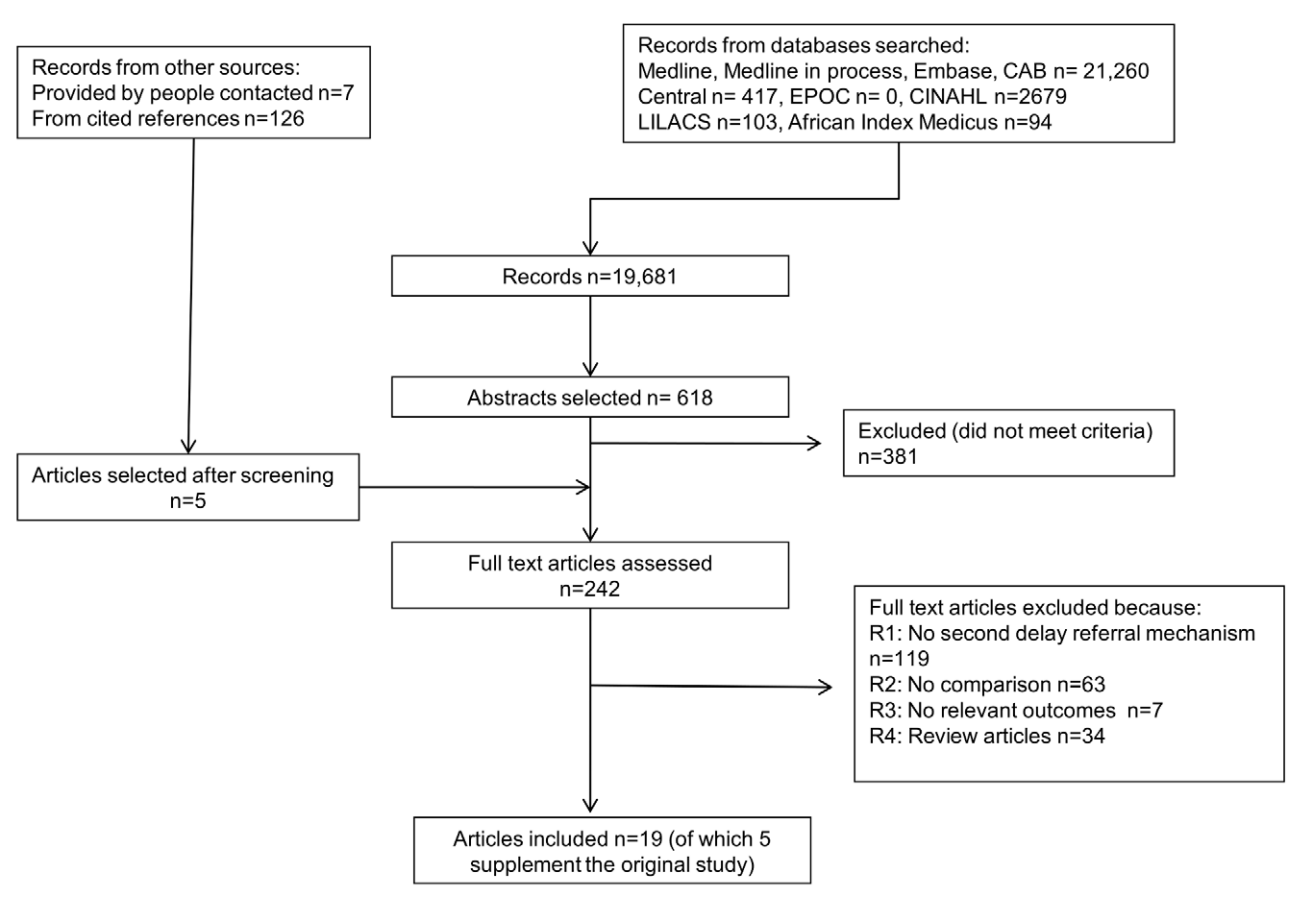
Study selection flow chart.

The search results are summarised in Figure 2. Nineteen papers met the study criteria, describing 14 different interventions (Table 2).

**Table 2:** Summary of characteristics of included studies.

Author/Country	Study Design	Setting	Level of Care at Referral Centre	Participants	Intervention Relevant to Phase II Delay	Other Interventions
**Organisational**						
Azad 2010/Bangladesh	Community-based cluster RCT, 9 clusters per arm; cluster sizes ranged from 15,441 to 35,110 population	Rural	NR	I 20,943 births; C 22,774 births	Women accompanied to facilities; Community emergency funds	Participatory women's groups; Training TBAs
Bailey 2002/Guatemala	Community-based controlled before and after study. Ten intervention clusters and 9 control, no information on cluster sizes	Rural (high-lands)	NR	I 1,819 women; C 1,699 women	TBAs enabled to surmount obstacles to transport including cost, institutional barriers; Health facilities encouraged to welcome TBA as birth companion	TBA training in detection, management and timely referral of complicated obstetric and neonatal cases
Bhutta 2008/Pakistan	Cluster RCT (a pilot study) 4 clusters per arm; cluster sizes ranged from 10,687 to 26,025 population	Rural	NR	I 2,932 live births; C 2,610 live births	Community groups establish emergency transport fund	Lady health worker training and TBA partnership; TBA newborn care training; Community health education
Hossain, 2006/Barbey 2001/Bangladesh	Community-based controlled before and after study. One intervention and one control district, population 153,000 and 183,000	NR	Health centre	I 713 births; C 796 births	Community groups establish emergency transport fund and to pay for hospital fees. Volunteers to accompany women or provide financial support	Upgraded facilities; Birth planning; Community support system; Volunteers to donate blood
Kumar 2008/India	Cluster RCT, 13 clusters per arm; cluster sizes ranged from 218 to 1,121 households	Rural	NR	I 1,522 live births; C 1,079 live births^a^	Community groups establish emergency transport fund	Community members provide newborn care, birth preparedness, clean delivery
Manandhar 2004/Nepal	Cluster RCT, 12 clusters per arm, cluster sizes ranged from 236 to 3,814 households	Rural	Health centre	I 3,036 pregnancies C 3,344 pregnancies	Community groups establish emergency transport fund	Participatory women's groups led by trained facilitators
**Structural**						
Alisjahbana 1995/Indonesia	Community-based cohort study with one intervention and one control subdistrict, population 40,000 and 87,000	Rural	Health centre and hospital	I 2,275 women; C 1,000 women	Birthing homes established; Radio communication and ambulance transportation	Physicians, midwives trained on case management; Home-based action records; TBA training; Improving women's knowledge
Brazier 2009/FCI 2007/Burkina Faso	Community-based controlled before and after study. One intervention and one control district, population 220,336 and 305,228	Rural	Health centre and hospital	I 2,554 women; C 2,859 women	Ambulance purchase; Radio call system	Obstetric care training; Provision of equipment and supplies; Quality assurance and management systems introduced
Chandramohan 1994, 1995/Zimbabwe	Cohort study of women who delivered at one hospital over 3 y, study population 4,488 women	Rural	Hospital	I 1,573 women; C 2,915 women	Building a maternity waiting home	None
Lonkhuijzen 2003/Zambia	Cohort study of women who delivered at one hospital over 6 mo, study population 510 women	Rural	Hospital	I 218 women; C 292 women	Maternity waiting home	None
Lungu 2001/Malawi	Community-based controlled before and after study, with 4 intervention and 6 control villages, size not stated	Rural	Health centre	I 41 women; C 53 women^a^	Bicycle ambulance placed in community	Community transport plan in other arm of study
Millard 1991/Zimbabwe	Cohort study of women who delivered at one hospital in 1 y, study population 854 women	Rural	Hospital	I 502 women; C 352 women	Antenatal village	None
Tumwine 1996/Zimbabwe	Cohort study of women who delivered at one hospital over years, study population 1,053 women	Rural	Hospital	I 280 women; C 773 women	Maternity waiting shelter	None
**Mixed**						
Fauveau 1991/Maine 1996/Ronsmans 2007 Bangladesh	Community-based controlled before and after study. Within one subdistrict, intervention and control areas were selected, population 47,808 and 51,468 during original 1991 study	Rural (flood plain)	Health centre and hospital	I 4,424; C 5,206	Boats, boatmen, helpers to accompany women. Referral chain with ambulances for onward referral	Nurse-midwives posted in outposts to work alongside community health workers and TBAs

a Studies with three arms, only the two relevant arms used for both studies.

C, control group I, intervention group; NR, not reported.

### Description of Studies

The studies were categorised using the EPOC taxonomy of interventions for practice change [9]. Fourteen interventions from 19 papers were included in this review. Of the 14 interventions, six were organisational in nature and seven structural. One study used structural and organisational mechanisms to enhance various types and levels of transport and the linkages between them. Interventions in a number of the included studies comprised several components, some of which were not related to referral.

The six organisational interventions involved surmounting obstacles to emergency transport, especially those of cost. All targeted women and community members including traditional birth attendants (TBAs) [10]–[16]. In five studies, community groups were organised to generate emergency funds for transport [10],[12]–[16]. Some of these studies indicated that the idea of emergency funds was generated through community mobilisation activities and was not pre-decided [10],[16], although the origin of the intervention was not so clearly described in other studies. The study in the rural highlands of Guatemala [11] comprised slightly different organisational mechanisms. In this study, TBAs accompanied women to health facilities, helped women surmount cost barriers, and were welcomed as birth companions in health facilities. None of the interventions solely addressed phase II delays and included other components such as improving integration between different health providers [12], education and awareness raising of complications [10],[15], and upgrading of facilities [13]. The organisational interventions included 4 RCTs. All were cluster randomised studies. In three of the RCTs [10],[15],[16], the authors of the original articles described their method of analysis, taking clustering into account and by intention to treat. One RCT [12] was a pilot study and details of how clustering was taken into account was not clear.

There were seven structural interventions that involved the use of maternity waiting homes, radios, and ambulances to overcome phase II delays [17]–[25]. Various forms of maternity or birthing homes were established, where pregnant women could stay, away from their own homes but close to a health facility. In Indonesia [17], the birthing home was located near a rural health centre and combined with several other health service improvements that addressed other phases of delay. The four sub-Saharan African studies described maternity waiting homes located close to a hospital [20]–[22],[24],[25] and were not combined with other interventions. The way in which the maternity waiting homes were used varied. Some women stayed because of risk factors or complications while others stayed by choice, even if there was no medical reason to do so. A transport intervention was studied in Malawi, where bicycles with an attached stretcher on wheels were located in the community [23]. In Burkina Faso and Indonesia, vehicles and radio communications were provided, but these were put into place alongside a variety of other interventions which affected phase I and II delays [17]–[19].

One intervention in Bangladesh included both organisational and structural characteristics [26]–[28]. Boats and ambulances were provided and in addition to these structural transportation elements, organisational traits were included such as assignment of boatmen and helpers to accompany women and improvement of linkages for onward referral to a district hospital and deployment of staff.

Four interventions were described in more than one paper. In the organisational intervention from Bangladesh [13],[14], the findings on effectiveness were found in the published paper [13], while the report [14] was used to provide details relating to the intervention and to confirm our understanding of the findings in the published paper. The same was done with the papers from Burkina Faso [18],[19]. In the case of the two papers on the maternity waiting home intervention in Zimbabwe [20],[21], one paper reported effects on maternal outcomes, and the other on perinatal mortality, so the two papers were used to extract information on different outcomes for the same intervention. In the mixed intervention from Bangladesh, the original paper [26] described the intervention and its effects on maternal mortality covering only the 3 y before and the 3 y after implementation of the intervention. The subsequent paper [28] reported on care for maternal complications and utilisation of services, using data from the original period and area. The third paper [27] presented maternal mortality data from the same geographical area in relation to the same intervention (which continued after the original study), but over a longer time period, covering the 11 y before and 7 y after the intervention.

All studies collected community-based data and provided information on outcomes at the population level, with the exception of the four maternity waiting home studies [20]–[22],[24],[25] and the study by Maine and colleagues [28], where the outcomes were in women attending hospital for delivery.

### Effects of Interventions on Health Outcomes

**Figure 3:**
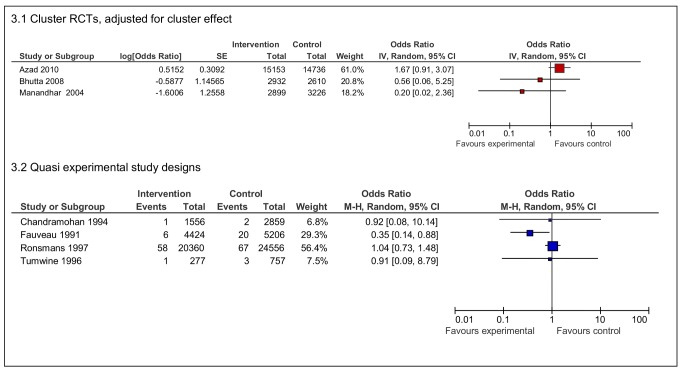
Effects of interventions on maternal deaths per live births.

Seven papers provided maternal mortality data (Figure 3). The three RCTs of community-targeted organisational interventions [10],[12],[16] showed varying effects, the sample sizes of the individual studies were not of adequate magnitude to measure this outcome with statistical confidence.

Of the quasi experimental studies reporting maternal mortality, the mixed organisational-structural intervention [26], which included transport improvements but also health staff deployment, showed a reduction in maternal mortality (odds ratio [OR] 0·35 95% CI 0·14–0·88), yet the study of the same intervention in the same site, which analysed mortality trends over a longer time, did not show the reduction to be sustained [27]. Two studies of maternity waiting homes [20],[25] had small numbers of maternal deaths confined to a hospital population and did not show reductions in maternal mortality. Maternal case fatality was only available from one study (forest plot not provided), which showed no difference between intervention and control groups (OR 0·68 95% CI 0·06–7·69) [28].

**Figure 4:**
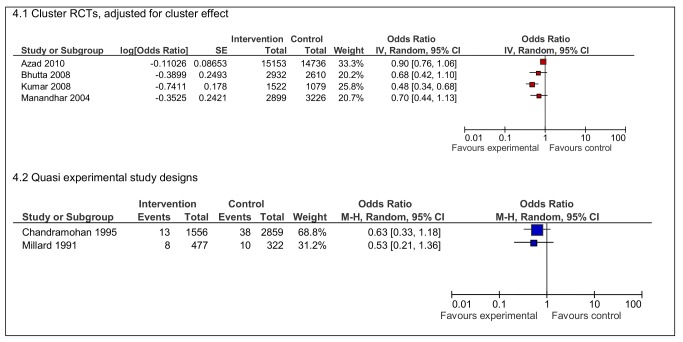
Effects of interventions on neonatal deaths per live births.

Neonatal deaths in four RCTs [10],[12],[15],[16] of organisational interventions were reduced, although the effects found were not equally strong (Figure 4). The largest effect was shown in the study from India (OR 0·48 95% CI 0·34–0·68) [15]. Neonatal deaths were also reduced in the quasi experimental studies of maternity waiting homes, although statistical significance was not demonstrated in these studies.

**Figure 5:**
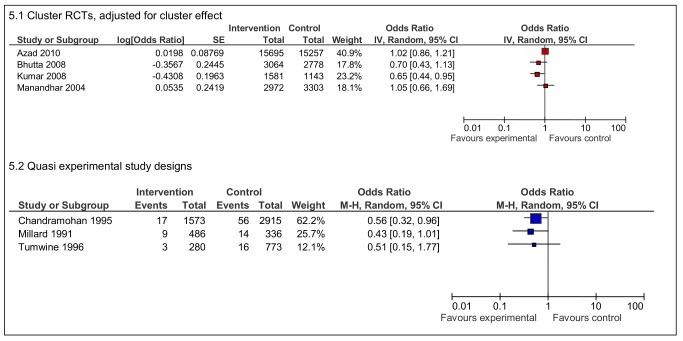
Effects of interventions on stillbirths per total live births.

There is less consistency in the data on stillbirths (Figure 5). Two RCTs demonstrate improvements in the intervention group [12],[15] but the ORs were close to one in the other two RCTs. Three quasi experimental studies on maternity waiting homes [21],[24],[25] demonstrated reductions in stillbirths, with one statistically significant result from Zimbabwe (OR 0.56 95% CI 0.32–0.96) [21].

### Other Effects of Interventions

Table 3 summarises the data available on outputs and intermediate effects of the various referral interventions. ORs could not be calculated for a number of studies because of a lack of data. Four studies: two organisational [15],[16] and two structural [17],[18], reported a higher proportion of deliveries with health professionals. One study [10] observed a lower rate in the intervention arm, but uptake of health services was noted to be higher in the control group even before the intervention.

**Table 3:** Summary table of intermediate outcomes and process measures, after intervention.

Referral Mechanism	Organisational Interventions Including Community Emergency Transport Funds and/or Support to Surmount Institutional, Cost Barriers						Structural Interventions, Including Maternity Waiting Homes, and/or Radios and/or Car or Bicycle Ambulances							Mixed, Organisational and Structural
	Azad 2010	Bhutta 2008	Kumar 2008	Manandhar 2004	Bailey 2002	Hossain 2006/Babey 2001	Alisjahbana 1995	Brazier 2009 and FCI 2007	Chandramohan 1994 and 1995	Lonkhuijzen 2003	Tumwine 1996	Millard 1991	Lungu 2001	Fauveau 1991/Maine 1996/Ronsmans 1997
Knowledge of intervention	NR	Unclear	Unclear	Unclear	NR	Unclear	NR	NR	Yes	Yes	NR	Unclear	Yes	Unclear
Referral rate of women with complication	NR	NR	NR	NR	I = 49%; C = 38%; OR 1.58 (0.90–2.80)	NR	I = 13%; C = 6%	Unclear	31%	I = 43%; C = 57%	NR	I = 59% C = 41%	NR	NR
Compliance with/use of intervention	NR	Unclear	NR	NR	I = 78%; C = 69%; OR 1.46 (0.65–3.27)	NR	I = 73%; C = 60%	NR	Yes^b^	Yes^b^	Yes^b^	Yes^b^	20%	NR
Travel time/distance	NR	NR	NR	NR	NR	NR	NR	Modal walking time >1 h'	150 m	“next to” ward	NR	2-min walk	5 km; 90 min	2–4 h
Costs	NR	NR	NR	NR	NR	US$ 0·03–0·06^a^	Pay “small” amount	“Low”	Free	US$9	Free	NR	US$ 0·1 per use	NR
Satisfaction with referral intervention	NR	NR	Unclear	Unclear	NR	NR	TBA reported loss of credibility	Unclear	Unclear	Unclear	Unclear	Unclear	Unclear	Unclear
Delivery in health facility	I = 15%; C = 16%	I = 31%; C = 13%; OR 3.01 (2.08–4.36)	I = 20%; C = 14%; OR 0.84 (0.54–1.32)	I = 7%; C = 2%; OR 3.56 (2.68–4.72)	I = 49%; C = 52%; OR 0.84 (0.54–1.32)	I = 21%; C = 5%	I = 12%; C = 4%; OR 3.24 (1.91–5.50)	I = 57%; C = 36%	NR	NR	NR	NR	I = 49%; C = 70%	NR
Delivery on route	NR	NR	I = 1.5%; C = 1.7%	NR	NR	NR	NR	NR	NR	NR	NR	NR	NR	NR
Delivery with health professional	I = 2%; C = 4%	NR	I = 27%; C = 20%; OR 1.49 (1.24–1.79)	I = 7%; C = 2%; OR 3.51 (2.66–4.63)	NR	NR	I = 13%; C = 5%; OR 6.27 (2.73–14.41)	I = 56%; C = 36%	NR	NR	NR	NR	NR	I = 13%
Met need^c^	NR	NR	NR	NR	NR	I = 40%; C = 12%	NR	NR	NR	NR	NR	NR	NR	NR

a Reported in some community groups only.

b Participants were selected on the basis of use or non-use of maternity waiting home.

c Proportion of complications seen to expected.

C, control group; I, intervention group; NR, not reported.

Data on health facility utilisation were available from nine studies. Six [12]–[18] showed increases in the proportion of deliveries in health facilities. The two RCTs that targeted the community reported improved health facility utilisation in intervention arms (OR 3.56 95% CI 2.68–4.72) [16] and (OR 3.01 95% CI 2.08–4.36) [12]. Of the remaining three studies that showed lower rates of delivery in health facilities in the intervention group, the reported effect of bicycle ambulances was most striking [23]. During the study, more women from villages without bicycle ambulances delivered in health facilities—70% in control villages compared to 49% of women in villages with bicycles. Furthermore, 22% of institutional deliveries during the study period came from villages provided with the bicycles and 42% from control villages (unpublished data). Of 20 instances where the bicycles were used, only four were for obstetric referrals, the rest for other medical conditions. The negative effect of the intervention on health facility utilisation was postulated to be due to a perception that bicycles brought unwanted attention to women during labour, so they preferred to walk to health facilities or deliver at home.

## Discussion

We focused our review on interventions that aimed to overcome phase II delays. We were not able to establish the effectiveness of referral interventions on maternal mortality or other intermediate or process outcomes such as uptake of care. Although we found some reductions in neonatal mortality and in stillbirths, inference of effect due to interventions that specifically overcome phase II delays is not possible due to design limitations of the individual studies.

Reduction in neonatal deaths were found in four South Asian RCTs of community mobilisation interventions, which included the generation of funds for transport, to overcome phase II delays [10],[12],[15],[16]. It was not possible to disentangle the effects of the phase II intervention with other components that addressed other types of delays or that improved care. The changes observed may have been a result of the other components, or may have occurred only if the various elements are combined. This finding confirms other reviews demonstrating the success of complex, community-based interventions in reducing neonatal mortality [29]–[31] but the contribution of referral to such improvements cannot be surmised.

There is a possibility that maternity waiting homes in sub-Saharan Africa may reduce stillbirth rates. Unlike the RCTs on community mobilisation, this group of studies on maternity waiting homes [20]–[22],[24],[25] focused on use of this structure enabling pregnant women to live physically closer to a health facility, so the interpretation regarding effect of the intervention may be less problematic. Nevertheless, the evidence is weak. The number of events recorded in these studies was small, the way the maternity waiting homes were used varied across and within studies, and bias likely in the studies, both because of the way the participants were selected as well as the restriction of the studies to facility-based data. Another systematic review of maternity waiting facilities was conducted in 2009 [32], concluding that there was insufficient evidence of effectiveness on maternal and perinatal outcomes, but stillbirths were not investigated as a specific outcome. It is plausible that being in a maternity waiting home will allow women to be seen more rapidly in the case of any untoward event. Assuming that rapid and effective action (such as monitoring of fetal wellbeing and/or expediting delivery) is taken, an intrauterine death could be averted. Maternity waiting homes are extensively used in many countries [33] despite the lack of evidence surrounding its effectiveness, so our finding provides an added rationale to support the conduct of well designed, primary studies on waiting homes. The mechanisms through which this intervention might work and the factors important for success (e.g., availability of surgery, or regular assessment of women staying in the maternity waiting home), could not be elicited from the studies included. Alongside questions of effectiveness, future research in this area should specify the intervention rigorously and explore pathways of effect.

Transport and communication interventions were another group of studies investigated in this review [17],[18],[23],[26]. Most comprised only one part of a multifaceted intervention that addressed other health service improvements, so few conclusions can be drawn regarding their effect on phase II delays. Common sense tells us that interventions that reduce travel time and link up the referral system are likely to be important; however, this review shows that what are apparently “good ideas” do need to be carefully assessed. The use of bicycle ambulances is one example. Many studies describe their utility [33], but this should not be assumed for all situations, as demonstrated by the adverse effect documented from the study in Malawi [23]. The relatively recent introduction of new technologies such as mobile phones may well improve referral. Although we came across studies describing the use of mobile phones and related technologies in obstetric referral [33], none fulfilled our study criteria. None of the included studies mentioned the use of mobile phones.

The outputs and intermediate effects of the various referral interventions were arranged in Table 3 in order of a postulated sequence of effect. For the referral intervention to have an effect, women would have to know about an intervention and/or be referred in the first instance. They would then have to comply with, or use, the referral intervention and subsequently overcome barriers they encounter, in order to reach “appropriate” care, which can be measured using proxies such as delivery at health facility, delivery with a health professional, or receipt of care during an obstetric complication (met need). Data were inconclusive and we were unable to use these indicators to trace or explain mechanisms through which the referral intervention might have worked. Two studies, from Guatemala and Indonesia, provided data on referral rate, compliance and utilisation. We were unable to ascertain whether the proportions reported in the Guatemalan study [11] shared the same denominators. The Indonesian study [17] found that 13% of pregnant women in the intervention group were referred. Of these, 73% complied, resulting in a statistically significant increase in deliveries with health professionals and in health facilities (Table 3), although perinatal mortality was not improved (OR 1.19 95% CI 0.81–1.75) (unpublished data).

Other reviews on referral interventions are available [5],[6],[33]–[36]. The three delays model [5] provided what is now a well-established paradigm for barriers in accessing emergency obstetric care. Two papers offer insights on factors likely to improve the implementation of referral interventions [6],[34]. Various technologies, transport, and physical communication options have been summarised [35]. Some reviews have improved understanding of barriers, such as transport and cost, that affect referral [6],[33],[36]. Underfunding of health systems has also been implicated in leading to inefficient referral [4]. Modelling techniques have predicted that maternal mortality decline will reach a threshold of less than 35% decline if access to emergency obstetric care is not provided, and that referral and transport strategies, alongside other interventions, could contribute to as much as an 80% reduction in maternal mortality [37]. Our systematic review provides a contribution to knowledge in this field by focusing on the quality of evidence and summarising estimates of effects. We faced considerable constraints because of the design and multi-component nature of some of the interventions. The heterogeneity we encountered (in terms of differences in interventions, selection of participants, study design, and reported effects) implied that a meta-analysis would not have provided coherent data nor helped with the difficulties encountered in disentangling the effects of complex interventions. It is possible that our search may not have exhaustively covered literature from low- and middle-income countries, especially if not in English [38],[39]. We believe it is unlikely we have missed key studies as discussions with colleagues and other groups studying obstetric referral and extension of our search to databases like LILACs and the African Index Medicus provided no new eligible studies.

Much remains unknown. Does function of the health system, terrain, or whether the intervention targets antenatal, intrapartum, or post partum periods matter when selecting which interventions to implement? The studies that met our inclusion criteria were set in rural areas—we can make no conclusions on urban settings or any other contextual factors. Investigating phase II delays in isolation may be an oversimplification as phase I and III factors will have effects on phase II and referral interventions work through complex mechanisms. However, the literature base on referral interventions as a whole is very large and until efforts are made to break down the various components into manageable parts, progress in understanding referral interventions cannot be made. Despite the wealth of literature describing means to improve women's access to maternity care during emergencies, know-how for effective implementation remains limited. The 19 out of over 600 potentially relevant studies that met the criteria for inclusion in our study, and the findings of other recent reviews on referral linkages [31],[33] is testimony to the low priority given to careful design of studies and good practice in monitoring and evaluation. In addition, studies found were not explicitly designed to explain how the effects of the referral interventions were achieved. Ten years ago, the tracking of individuals who have been referred was recommended as a way to address this gap [34],[36], but little new knowledge has been generated in this area. Now that a start has been made in appraising the referral literature systematically, studies to investigate causal pathways and mechanisms of effect are necessary to understand how the interventions work as one part of a chain reliant on other components of the health system, rather than in isolation.

### Conclusion

The limitations inherent in the studies included in this review mean that findings should be interpreted with caution. We found that complex, community-targeted interventions reduce neonatal mortality but not how the referral components contributed. The reduction in stillbirths observed in studies of maternity waiting homes makes this a potentially promising intervention that needs further investigation. While continuing to invest in implementing referral interventions within maternal and newborn health programmes, we urge health planners to ensure that the interventions are rigorously monitored and evaluated, and operations research studies designed with controls and comparisons. There should be awareness that referral interventions may have adverse effects. Future research should aim to understand how the interventions work and why, by using methods that provide understanding of causal pathways and mechanisms of action.

## Supporting Information

Text S1 **PRISMA checklist.**
(DOC)

Text S2 **Review protocol.**
(PDF)

Text S3 **Search strategy.**
(DOC)

## Abbreviations

OR: odds ratio
RCT: randomised controlled trial
TBA: traditional birth attendant
