# Supplementary material for: The Effectiveness of Emergency Obstetric Referral Interventions in Developing Country Settings: A Systematic Review
Source: PLoS Med. 2012 Jul 10;9(7):e1001264. doi: 10.1371/journal.pmed.1001264 (PMC3393680; doi:10.1371/journal.pmed.1001264)
Supplement: Text S2 — Review protocol. (PDF) [file pmed.1001264.s002.pdf]

DFID Systematic Review Program
(PO 40031891)

PROTOCOL FOR PEER REVIEW

Revised post review by DFID, Alain Mayhew and Susan Murray 31st July 2010

**Main title**	What kinds of policy and programme interventions contribute to reductions in maternal mortality?
**Sub title**	The effectiveness of primary level referral systems for emergency maternity care in developing countries.
**Review group**	The review team comprises three co-investigators and a research assistant:<br><br>• Julia Hussein (JH) is the principal investigator and a public health obstetrician. At the University of Aberdeen, she conducts evaluation research on maternal mortality reduction strategies in developing countries as part of the global research initiative for maternal mortality reduction called Immpact.<br><br>• Stephen Munjanja (SM) is a consultant obstetrician at Harare Hospital in Zimbabwe. He has an international profile as a researcher with interests in maternal and neonatal mortality measurement, randomised trials, population based studies and assessment of obstetric care services.<br><br>• Margaret Astin (MA) is an experienced systematic reviewer who has been particularly involved in reviews of complex public health interventions, health care systems, radiotherapy for cancer and diagnostic imaging test performance. She has conducted Cochrane reviews, has held positions as an information scientist and produces guidelines and reviews for UK National Health Service.<br><br>• Lovney Kanguru (LK) is the research assistant with an MSc in International Health and Management. She is based at the University of Aberdeen.<br><br>JH and SM will be the individuals responsible for appraising the content of the systematic review. MA will provide the expertise in systematic review methods and information retrieval. The co-investigators will be supported by LK.
**Section**	PROCOTOL
**Authors IN ORDER OF CREDIT**<br><br>**(Please include first and surnames, institutions. Include titles – Dr, Prof – if you want them to be used.)**	Dr. Julia Hussein (Principal Investigator)<br>Senior Clinical Research Fellow Immpact<br>Scientific Director Ipact<br>University of Aberdeen<br>Health Sciences Building<br>Foresterhill, Aberdeen AB25 2ZD<br>Tel 01224 554474/559736 Fax 555704

	Email j.hussein@abdn.ac.uk<br><br>Margaret Astin (Co-investigator)<br>Systematic reviewer<br>Departments of Social Medicine and Primary Health Care<br>University of Bristol<br>Tel 0117 331 3841<br>Email m.p.astin@bristol.ac.uk<br><br>Dr. Stephen Munjanja (Co-investigator)<br>Senior Lecturer<br>Department of Obstetrics and Gynaecology<br>College of Health Sciences, University of Zimbabwe<br>PO Box A178, Avondale, Harare, Zimbabwe<br>Tel 263 4 253 724 , 263 4 791 631 Fax 263 4 723 711<br>Email Spmunjanja@africaonline.co.zw<br><br>Lovney Kanguru (Research Assistant)<br>University of Aberdeen<br>Health Sciences Building<br>Foresterhill, Aberdeen AB25 2ZD<br>Tel: 01224 552764<br>Email l.n.kanguru@abdn.ac.uk
**EPPI-Centre reference number**	n/a
**Month/year of publication**	n/a
**This report should be cited as…**	Protocol for systematic review on referral systems
**Contact details**<br><br>**(address, phone number, email)**	Dr. Julia Hussein (Principal Investigator)<br>Senior Clinical Research Fellow Immpact<br>Scientific Director Ipact<br>University of Aberdeen<br>Health Sciences Building<br>Foresterhill, Aberdeen AB25 2ZD<br>Tel 01224 554474/559736 Fax 555704<br>Email j.hussein@abdn.ac.uk
**Institutional base**	University of Aberdeen
**Review Group**<br><br>**(with institutions)**	Alain Mayhew of the University of Ottawa<br>Susan Murray of Kings College London
**Advisory group**<br><br>**(with institutions)**	Not required, same as review group
**Conflicts of interest (if any)**	None known. The principal investigator (JH) was involved in the development and implementation of referral interventions in Malawi between 1997-2000 as a DFID technical adviser. JH is involved in editing a book on maternal health which contains a chapter on referral systems (which is authored by SM).
**Acknowledgements**	DFID for funding the protocol.

1. Background

1.1 Aims and rationale for review

Current estimates of maternal deaths in the developing world are unacceptably high. The maternal mortality ratio in developing countries was estimated to be 450 per 100,000 live births in 2005 by the World Health Organisation, (Hill et al 2007) although recent studies have reported lower estimates of 251 per 100,000 live births for 2008 (Hogan et al 2010). Neonatal mortality in developing countries is 31 per 1000 live births (UNICEF 2009). The health of women and their children are interlinked and many factors that contribute to the high levels of mortality are common to mothers and the newborn child. These factors are wide ranging and include the low status of women, cultural and economic barriers, poor nutrition, conditions such as HIV/AIDS or malaria, lack of appropriate health care facilities and poor access to skilled and emergency obstetric care. Many obstetric emergencies such as haemorrhage and obstructed labour are unpredictable and can have catastrophic consequences within a short period of time. For example, a serious post partum haemorrhage can lead to death of a woman in less than 2 hours and the unborn fetus may succumb much earlier (AbouZahr 1998). In the poorest countries, two thirds of women deliver at home, far from emergency services or without access to a health professional (UNICEF 2009). Maternal and neonatal deaths could therefore be prevented if functional referral systems were in place to allow pregnant women to reach the appropriate health services when complications occur.

The aim of this review is to look more closely into interventions designed to address delays in referral and to assess the effects of the relevant (primary level) referral system interventions. The delays of interest in this review are those experienced between making the decision to seek care and in reaching the appropriate level of facility. These are known as Phase II delays (Thaddeus & Maine 1994, see section 1.2). We will focus on referral to access emergency obstetric care from home to basic level health facilities (health centres) and from health centre to hospital (but not referral between hospitals). We expect to include a wide range of different types of interventions, such as: means of improving transport and communications, technologies for improving access to specialist skills, strengthening linkages between social networks training for referral and outreach interventions to bring care closer to the community level.

1.2 Definitional and conceptual issues

The three delays model (Thaddeus & Maine 1994) provides a conceptual framework of the factors influencing the timely arrival to appropriate care in obstetric emergencies. These are (i) delays in the recognition of the problem and the decision to seek care in the household, (ii) delays in reaching the appropriate facility, and (iii) delays in the care received once the woman reaches the facility. Although distance and cost of care are among the major factors in the decision to seek care, or Phase I delay, the quality of care provided by facilities and the communities' perception of the quality may also influence the decision. The second delay of the model, or Phase II, is concerned with the delay in arrival at the health facility, after the decision to seek care has been made. This phase is determined by the distribution and location of health facilities (and health professionals) which are equipped to deal with emergency obstetric and neonatal care, as well as the availability and costs of transportation and communication systems to reach facilities. These delays are most common and severe in rural areas, where health professionals may not be available, transport and communication systems limited, and road conditions changeable, depending on the season. The third, or Phase III, delay occurs at the facility level where delays in various aspects of care delivery such as staff shortages, staff attitudes and skills, a lack of functioning equipment, unreliable drug and blood supplies, and inadequate management structures, individually or together reduce the quality of care available.

Figure 1 provides a schema of the conceptual framework underlying this review.

FIGURE 1: CONCEPTUAL FRAMEWORK FOR THE REVIEW (Adapted from Thaddeus & Maine 1994)

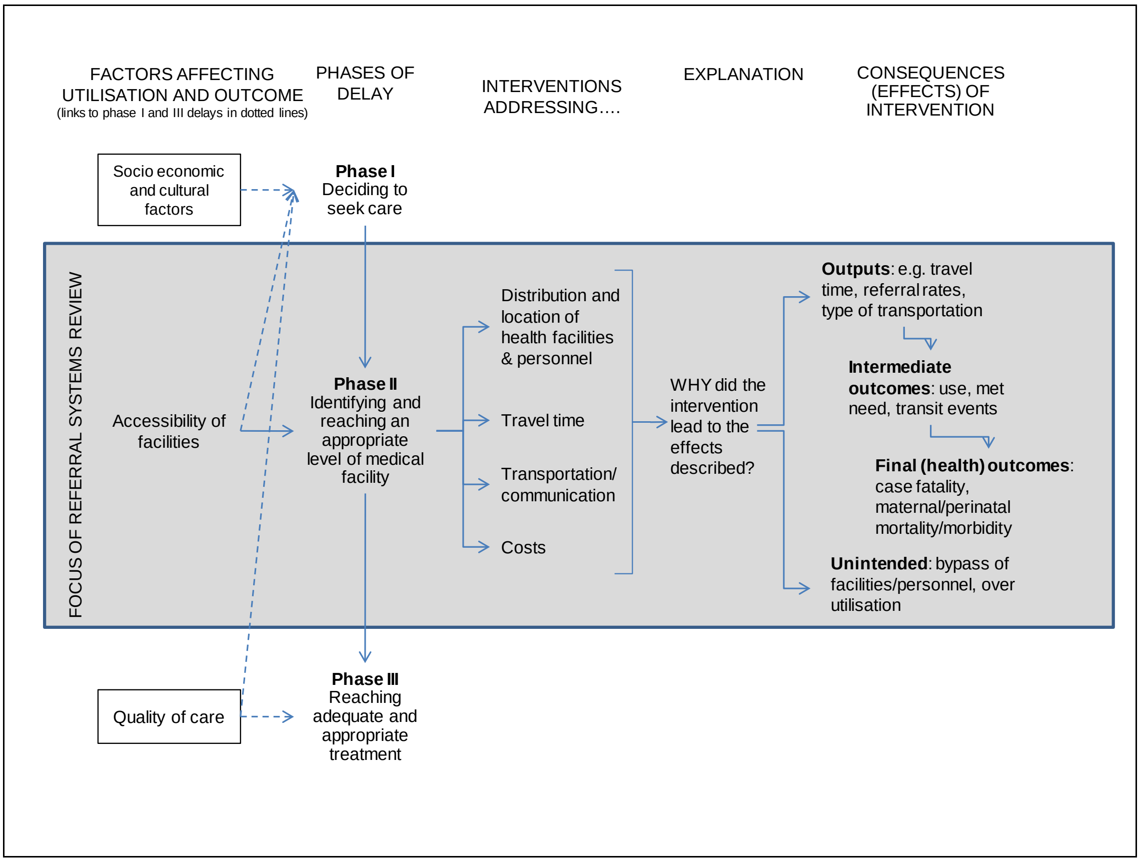

1.3 Policy and practice background

Maternal and neonatal deaths could be prevented if functional referral systems were in place to allow pregnant women to reach the appropriate health services when complications occur. Various interventions to improve referral systems are currently recommended. The evidence related to the effects of these interventions is challenging to interpret due to the diversity of studies, different measures of effects and variation in quality of studies. The prerequisites for a functioning maternity referral system are well described in the literature, however there is scarce evidence of the effects of systems implemented in practice. Given this situation, the findings of this review will provide evidence to inform policy and programme decisions, so that “best value” will be obtained from the investment made in referral interventions.

1.4 Research background

We completed a preliminary scoping of the literature to assess the size and quality of the existing evidence base. Four facets were used to search for relevant literature: terms for emergencies in health care systems, terms for referral, terms for developing countries, and terms for all types of study designs. Over 6,000 abstracts were identified. By excluding reviews, editorials, debates, papers with no focus on obstetric referral and clinical studies we anticipate that a few hundred papers will need to be examined in further detail. We expect to find few studies of interventions evaluated using randomised controlled trials and few studies showing effects on health outcomes.

Two reviews have summarised studies on referral systems relevant to developing countries. Murray (2006) conducted a literature review identifying key requisites for maternity referral systems in developing countries. The findings suggest that successful referral systems are likely to be informed by population needs, adequately resourced referral centres, active

collaboration between referral levels and across sectors, formalised communication and transport arrangements, specific protocols for referrer and receiver, provider performance monitoring, affordable costs and policy support. The review by Krasovec (2004) considered transportation and communication for obstetric emergencies, and described the findings from a number of mostly low quality studies. A range of transport options were assessed, these were usually integrated with communication strategies such as physical communication systems, e g, radio, funding schemes, or intermediate schemes such as maternity waiting homes and birth and emergency preparedness plans. Recommendations were based on translating available technologies into routine practice, and that the introduction of a technological improvement is not introduced in isolation, rather it is usually one aspect of a multifaceted approach. These reviews highlight the complexities of strengthening health systems, and imply that simple interventions may not be enough in isolation.

Examples of interventions described in the literature include:

- Upgrading referral centres to provide comprehensive services. The upgraded services have improved provision of blood and drug supplies, 24 hour health care services or surgical facilities (Sabitu et al 1997, Bailey et al 2002, Dwivedi 2002)
- Training/awareness raising of various types of health workers and community members (Jokhio et al 2005, Manandhar et al 2004, Alisjahbana et al 1995, Bailey et al 2002)
- Use of technologies such as telephones, adaptations to vehicles or telemedicine interventions (Hofman et al 2008, Lungu et al 2001, Geerts et al 2004)
- Various financing and incentive schemes to assist transfer during an emergency (Hossain et al 2006, Barbey et al 2001, Essien et al 1997)
- Provision of first aid such as anti-shock garments, drugs for community use and life saving skill enhancement (Miller et al 2010, Mavalankar et al 2009)
- Clinical guidelines, including monitoring at risk patients for potential complications and improvement of recording systems (McCaw-Binns et al 2004, Danquah et al 1997, Kongnyuy et al 2008)
- Organisational changes such as introduction of intermediate level maternity units (including maternity waiting homes), emergency response teams, referral centres, outreach clinics (Fahdhy et al 2005, Foord et al 1995, Chandramohan et al 1995).

1.5 Objectives

The overall objective is to assess the effects of referral system interventions for timely referral to higher levels of care or emergency care in developing countries. Referral of the pregnant or postpartum woman is the focus of interest. This will have implications on the wellbeing of the mother, unborn baby and the newborn so maternal and perinatal outcomes are of interest, along with process indicators of service utilization, timeliness and delays.

The specific objectives are:

- To compare the effects of different referral interventions.
- To compare the effects of interventions disaggregated by subgroups and settings, categorised according to:
    - Levels of maternal mortality
    - Rural, urban or intermediate settings
    - Geographical terrain
    - Different periods of pregnancy (antenatal, intrapartum, postpartum)
    - Wealth
    - Functionality of the health system
    - Origin and initiator of intervention
- To identify factors explaining the effects of the various interventions.

2. Methods used in the review

2.1 User involvement

2.1.1 Approach and rationale

We aim to reach a variety of local, national and international stakeholders using the outputs of the review (the interim and final reports, policy and web-based summaries, open access academic publications and inclusion of lists of studies in an evidence database). The audiences likely to use the findings of the study include:

- Local non government organisations, especially those working on improvement of referral, community members, private and public health providers
- Developing country governments and their development partners (at district, state, national levels), especially individuals involved in maternal and neonatal mortality reduction programmes and health systems improvements
- International organisations, donor groups, professional and academic bodies involved in promoting maternal health

To maximise interest in, and uptake of, the findings of the study, we aim to target our communication outputs to groups (e.g. Partnership for Maternal, Newborn and Child Health, The Maternal Health Task Force, FIGO, UN organisations, donor organisations, international NGOs) which are networked or linked to the audiences listed above. We will also position the timing of specific communication activities to link up with key global and national events (such as presentations at themed symposiums or conferences) and where opportunities arise to influence country planning cycles and initiation of new maternal and neonatal health programmes.

2.2 Identifying and describing studies

2.2.1 Defining relevant studies: inclusion and exclusion criteria

<u>Participants</u>
Pregnant and post-partum women suffering from an obstetric complication, who are referred as an emergency, from the community or from a primary care centre to a facility where comprehensive emergency obstetric care is available. The conditions which require referral that are potentially relevant include:

- Obstetric complications and emergencies, including interventions used to stabilize the patient before arrival at the facility
- Participants who, to assure birth with a skilled attendant, or are at risk of complications in child birth, utilize facilities which reduce travel time or distance to the referral centre. Examples would include maternity waiting homes and voluntary relocation of women.

Exclusions: Referrals of the newborn baby, women with non-maternity related conditions or non-emergency referral cases, women who are being transferred between hospitals.

<u>Types of interventions</u>
All interventions to improve emergency referral in the antenatal period and detection and referral of potential complications during the intrapartum or postpartum period (up to and including 42 days after delivery); and which are relevant to improving referral and referral systems will be relevant to this review. These may lie on a continuum of single interventions (e.g. training, transport, incentives) to combinations of interventions or organisational changes (e.g. providing new or upgraded of referral facilities, improving linkages between different referral levels). Interventions must be critical or important to the second (Phase II) delay. In studies where health system strengthening has been implemented such as improving the quality of care at the referral centre, then this will be taken into account in the discussion. A proposed classification of interventions is provided in section 2.3.2.

Exclusions: Interventions to improve Phase I and III delays (Thaddeus & Maine 1994), including those that change decision making, transfers between tertiary care centres, and non-emergency referrals.

Types of measures
In accordance with our conceptual framework (Figure 2) the types of measures we will review lie along a pathway of direct effects of the intervention (outputs of intervention) to the final desired outcome of improved mortality and morbidity, as follows:

- Output measures will include travel time, referral rates, type of transportation or communication, direct and indirect costs (payments for transport, health facility fees, loss of income), women's knowledge of pregnancy or postpartum complications and satisfaction with intervention
- Intermediate outcomes include indicators of utilisation levels, met need (proportion of complications seen to expected complications)
- Final outcomes are health outcomes such as maternal and neonatal mortality and morbidity including near misses, stillbirths, live births, complication rate and case fatality rates. These will be included where recorded.

Exclusions: Neonatal deaths after the first week of life, as these tend not to be related to maternal complications.

Settings
All developing countries are potentially eligible for inclusion. Developing countries will include low income, lower middle income and upper middle income economies as classified by the World Bank (http://data.worldbank.org/about/country-classifications/country-and-lending-groups).

Exclusions: Refugees, war zones, mass casualties. Although we acknowledge that the inclusion of special settings in developed (high income) countries such as rural Australia may provide relevant information, we have not included these as resource availability and the existing health system infrastructure may influence the feasibility and effectiveness of the interventions being considered.

Study design:
Randomised or quasi-randomised studies with a control or comparison group, non-randomised prospective studies with a comparison group, controlled before-after studies, and interrupted time series (ITS) of referral systems for emergency maternity care from published and grey literature.

Exclusions: Studies without a comparison group.

Our initial literature search has already identified studies such as Manandhar et al 2004, Jokhio et al 2005 and Hossain et al 2006 which fulfil the criteria above, so we believe there will be sufficient material to inform the review.

2.2.2 Identification of potential studies: Search strategy

The search strategy listed below will be run in MEDLINE (1950-to current) on the OVID platform. Search terms are detailed in Annex 1.

We will adapt this search strategy for EMBASE (January 1985 to current), CINAHL (1985 to current), the Cochrane Central Register of Controlled Trials (CENTRAL), and LILACS (1985 to current) by selecting appropriate MeSH and/or keywords from their respective thesauri.

The Cochrane Effective Practice and Organisation of Care (EPOC) Register will be searched from 1985 to current.

We will also search the POPLINE, Reproductive Health Gateway and id21 databases and departmental bibliographic databases, using relevant keywords available on the search

interfaces. More specific databases, Africa Journals Online, African Health Line, India Med and Institute of Tropical Medicine will also be searched using relevant terms.

The grey literature and reports from relevant programmes will also be considered. Some contacts will be made and Internet sites checked including CEDPA, WHO, World Bank, JHPIEGO, John Snow Inc, Safe Motherhood Initiative, The White Ribbon Alliance, USAID, UNICEF, UNFPA, Riders for Health, Save the children, World Vision, CARE International and others

Studies and grey literature reports identified from reference lists, related systematic reviews and personal contacts will be considered for relevance.

No language restrictions will be applied, relevant papers will be translated if required (Formal translation costs are not expected as few papers are expected to be found in languages other than English and for these papers, Immpact has access to scientists with Spanish, Portuguese and French language skills).

Citations identified from electronic searches will be downloaded to a Reference Manager database. Titles and abstracts will be screened for relevance against the inclusion criteria independently by at least 2 reviewers from within the review team. Full copies of studies that may meet the inclusion criteria will be obtained. Reference lists of relevant systematic reviews, narrative reviews and of included studies will also be screened for potentially relevant primary studies and reports. Disagreements about inclusion will be resolved by discussion, and where unresolved, a third opinion will be sought. Authors will be contacted for further information or relevant unpublished data where necessary.

2.2.3 Screening studies: applying inclusion and exclusion criteria

Inclusion and exclusion criteria will be applied successively to (i) titles and abstracts and (ii) full reports. Full reports will be obtained for those studies that appear to meet the criteria or where we have insufficient information to be sure. These reports will be entered into a second database. The inclusion and exclusion criteria will be re-applied to the full reports and those that do/did not meet these initial criteria will be excluded. A log of excluded studies with reasons for exclusion will be kept.

2.2.4 Characterising included studies

We envisage a number of ways of classifying interventions. One way would be by the type of person (health professional, community worker, lay person) on which the intervention is focused; another by the place (home, health post, health facility) the intervention takes place; or by the characteristic of the intervention (educational, organisational or financial) as has been done in other studies of (non maternity) referral systems (Akbari et al 2009). We intend to use the taxonomy of interventions recommended by Davies et al (2000) of professional, financial, organisational, patient-oriented, structural and regulatory, because these are likely to provide a classification that allows clear identification for changes in practice. The suitability of this classification will be reassessed during the analysis.

2.2.5 Identifying and describing studies: quality assurance process

A data extraction form will be developed and the data extracted relevant to study setting, design, participants, type of intervention (including a description of simple and complex interventions), methodological parameters and outcomes assessed. The form will be piloted by two review team members using a representative sample of included studies in order to make any necessary amendments. The need for change will be discussed between the two individuals, consensus reached and modifications to the form made.

Data will be extracted by one of four reviewers and checked by a different reviewer. Any disagreements will be resolved by discussion between the reviewers, and if necessary an arbitrator within the review team. Where data are not available in the published report, authors will be contacted for missing information. Where relevant data is not available or the author not contactable, then the data will be assessed qualitatively.

2.3 Methods for synthesis

2.3.1 Assessing quality of studies

Methodological quality of included studies will be assessed considering study design, selection bias, confounders, blinding of outcome assessors, data collection methods, withdrawals and dropouts, and integrity of interventions. The EPOC quality criteria (http://epoc.cochrane.org/epoc-resources-review-authors) will be used as a guide. As a range of study designs are likely to be included and the interventions multi-faceted, assigning overall rating may not be straightforward. Two reviewers will assess quality of studies independently. Any disagreements in quality ratings will be resolved by discussion between the reviewers, and if necessary an arbitrator.

2.3.2 Overall approach to and process of synthesis

We intend to use a thematic synthesis which will classify interventions according to the various anticipated categories described in section 2.2.4.

2.3.2.1 Selection of studies for synthesis (if not all studies that are included in the synthesis)

All studies will be included in the synthesis, according to the inclusion and exclusion criteria identified in previous sections.

2.3.2.2 Selection of outcome data for synthesis

The outcome data used will include the outputs of the intervention, intermediate outcomes and final (health) outcomes, in accordance with our conceptual framework (Figure 1).

2.3.2.3 Process used to combine/ synthesise data

It is anticipated that because of the diversity of studies, contexts and complexity of interventions that the majority of data will be assessed by a qualitative summary, using the quality of studies and size and direction of effects. For the primary outcomes appropriate graphical methods of displaying the findings will be used where appropriate (e.g. bubble plots or box and whisker displays).

The primary analyses will examine the output, intermediate and final outcome measures. These will be tabulated and the effects of contextual factors explained qualitatively using the quality of studies, size and direction of effects.

Secondary analyses will compare and if possible, disaggregate data from studies with contextual factors and process measures using tables and a qualitative description of findings relating to the following subgroups:

- Burden of maternal mortality
- Rural, urban, semi-urban, and intermediate settings (maternity waiting homes, outreach clinics)
- Geographical terrain
- Different participant subgroups, e.g. antenatal, intrapartum, postpartum periods or by wealth quintiles
- Initiator of intervention, e.g. private for profit, non governmental, government
- Functionality or investment in health system e.g. weak or strong
- Origin of intervention – e.g. research driven design, development through practice, wider scheme with strong monitoring

Where participants are randomized by clusters, e g, villages or clinics, without accounting for clustering in the analysis, we will re-analyze to minimise unit of analysis errors. If this is not possible the point estimate will be reported.

We will explore heterogeneity using tables and bubble plots where possible. Potential effect modifiers include facility upgrades and co-interventions that influence the referral process. Confounding effects may be looked at in a limited way if there is sufficient data through

subgroup or sensitivity analyses; discussion; or meta-regression techniques if possible. Other means of analysing and summarising data quantitatively have been used where eligible studies exhibit considerable heterogeneity (Shojana et al 2010) and these methods of analysis will be considered as the relevant articles are identified.

Our initial proposal did not envisage complex statistical analysis so no funds have been budgeted for a statistician. However, we have been encouraged in the review process to consider the possibility of further quantitative summaries and this decision will be taken when preliminary results of the review are available. Statistical assistance will be available from within the University of Aberdeen although some top up funding may be required to secure the time of a statistician. We understand DFID is in support of such additional input, but this will require further discussions at a later stage of the review.

2.4 Deriving conclusions and implications

We will use a participatory means of drawing inferences and conclusions from our results. In order to do so, preliminary findings will be synthesised and interpreted as an interim report. The interim report will result from debate and discussion initially within the review team. We will then share this interim report with a small, purposively selected group of individuals drawn from our target audience (see section 2.1.1) who will be asked (a) if they agree or disagree with our interim conclusions and recommendations (b) to suggest their conclusions, if different/new (c) to comment on the policy and practice implications of the report as a whole from their perspective. In addition, the two named reviewers will be asked to review the report. The collated comments will be discussed by the review team and incorporated within the report where the review team agrees is appropriate.

References

AbouZahr C: Antepartum and Postpartum hemorrhage. Volume Chapter 4. First edition. Boston (United States of America) Geneva (Switzerland): Harvard School of Public Health on behalf of the World Health Organisation and the World Bank; 1998

Akbari A, Mayhew A, Al-Alawi MA, Grimshaw J, Winkens R, Glidewell E et al Interventions to improve outpatient referrals from primary care to secondary care Cochrane Database of Systematic Reviews Issue 4, Art No: CD005471 DOI: 10.1002/4651858.CD005471.pub2.

Alisjahbana, A., Williams, C., Dharmayanti, R., Hermawan, D., Kwast, B. E., & Koblinsky, M. 1995, 'An integrated village maternity service to improve referral patterns in a rural area in West-Java', International Journal of Gynaecology & Obstetrics, vol. 48 Suppl, p. S83-S94.

Bailey, P. E., Szászdi, J. A., & Glover, L. 2002, 'Obstetric complications: Does training traditional birth attendants make a difference?', Revista Panamericana de Salud Publica/Pan American Journal of Public Health, vol. 11, no. 1, pp. 15-23.

Barbey, A., Faisel, A.J., Myeya, J., Stavrou, V., Stewart, J., Zimicki, S. 2001. Dinajpur SafeMother Initiative. Final Evaluation Report. CARE.

Chandramohan D, Cutts F, Millard P. 1995 The effect of stay in a maternity waiting home on perinatal mortality in rural Zimbabwe. J Trop Med Hyg. 98(4):261-7.

Danquah JB, Appah EK, Djan JO, Ofori M, Essegbey IT, Opoku S. 1997 Improving recordkeeping for maternal mortality programs, Kumasi, Ghana. The Kumasi PMM Team. Int J Gynaecol Obstet. 59 Suppl 2:S149-55.

Davies HTO, Nutley SM et al 2000 EPOC taxonomy of interventions aimed at achieving practice change. What works? Evidence based policy and practice in public services. Bristol: The policy press.

Dwivedi H, Mavalankar D, Abreu E, Srinivasan V. 2002 Planning and implementing a program of renovations of emergency obstetric care facilities: experiences in Rajasthan, India. Int J Gynaecol Obstet. 78(3):283-91.

Essien E, Ifenne D, Sabitu K, Musa A, Alti-Mu'azu M, Adidu V, Golji N, Mukaddas M. 1997 Community loan funds and transport services for obstetric emergencies in northern Nigeria. Int J Gynaecol Obstet. 59 Suppl 2:S237-44.

Fahdhy, M. & Chongsuvivatwong, V. 2005, 'Evaluation of World Health Organization partograph implementation by midwives for maternity home birth in Medan, Indonesia', Midwifery, vol. 21, no. 4, pp. 301-310.

Foord, F. (1995) Gambia: evaluation of the mobile health care service in West Kiang district. World Health Statistics Quarterly, vol. 48, pp. 18-22.

Garritty C, Tsertsvadze A, Tricco AC, Sampson M, Moher D, 2010 Updating Systematic Reviews: An International Survey. PLoS ONE 5(4): e9914. doi:10.1371/journal.pone.0009914.

Geerts, L., Theron, A. M., Grove, D., Theron, G. B., & Odendaal, H. J. 2004, 'A community-based obstetric ultrasound service', International Journal of Gynaecology & Obstetrics, vol. 84, no. 1, pp. 23-31.

Hill, K., et al., Estimates of maternal mortality worldwide between 1990 and 2005: an assessment of available data. Lancet, 2007. 370(9595): p. 1311-9.

Hofman JJ, Dzimadzi C, Lungu K, Ratsma YEC, Hussein J. (2008) Motorcycle ambulances for referral of obstetric emergencies in rural Malawi: Do they reduce delay and what do they cost? International Journal of Gynaecology and Obstetrics 102: 191-197.

Hogan, M.C., et al., Maternal mortality for 181 countries, 1980-2008: a systematic analysis of progress towards Millennium Development Goal 5. Lancet, 2010. 375(9726): p. 1609-23

Hossain, J. & Ross, S. R. 2006, 'The effect of addressing demand for as well as supply of emergency obstetric care in Dinajpur, Bangladesh', International Journal of Gynecology & Obstetrics, vol. 92, no. 3, pp. 320-328.

Jokhio, A. H., Winter, H. R., & Cheng, K. K. 2005, 'An intervention involving traditional birth attendants and perinatal and maternal mortality in Pakistan', New England Journal of Medicine, vol. 352, no. 20, p. 19.

Kongnyuy EJ, Leigh B, van den Broek N. 2008 Effect of audit and feedback on the availability, utilisation and quality of emergency obstetric care in three districts in Malawi. Women Birth. 21(4):149-55.

Lungu K, Kamfose V, Hussein J, Ashwood-Smith H (2001). Are bicycle ambulances and community transport plans effective in strengthening obstetric referral systems in Southern Malawi? Malawi Medical Journal 12(2): 16-18.

Manandhar, D. S., Osrin, D., Shrestha, B. P., Mesko, N., Morrison, J., Tumbahangphe, K. M., Tamang, S., Thapa, S., Shrestha, D., Thapa, B., Shrestha, J. R., Wade, A., Borghi, J., Standing, H., Manandhar, M., Costello, A. M., & Members of the MIRA Makwanpur trial team. 2004, 'Effect of a participatory intervention with women's groups on birth outcomes in Nepal: cluster-randomised controlled trial', Lancet, vol. 364, no. 9438, pp. 970-979.

Mavalankar D, Callahan K, Sriram V, Singh P, Desai A. 2009 Where there is no anesthetist--increasing capacity for emergency obstetric care in rural India: an evaluation of a pilot program to train general doctors. Int J Gynaecol Obstet 107(3):283-8.

McCaw-Binns, A. M., Ashley, D. E., Knight, L. P., MacGillivray, I., & Golding, J. 2004, 'Strategies to prevent eclampsia in a developing country: I. Reorganization of maternity services', International Journal of Gynecology & Obstetrics, vol. 87, no. 3.

Miller S, Fathalla MM, Youssif MM, Turan J, Camlin C, Al-Hussaini TK, Butrick E, Meyer C. 2010 A comparative study of the non-pneumatic anti-shock garment for the treatment of obstetric hemorrhage in Egypt. Int J Gynaecol Obstet. 109(1):20-4.

Moher D, Tsertsvadze A, Tricco A, Eccles M, Grimshaw J, Sampson M, Barrowman N. When and how to update systematic reviews. Cochrane Database of Systematic Reviews 2008, Issue 1. Art. No.: MR000023. DOI: 10.1002/14651858.MR000023.pub3.

Opoku SA, Kyei-Faried S, Twum S, Djan JO, Browne EN, Bonney J. 1997 Community education to improve utilization of emergency obstetric services in Ghana. The Kumasi PMM Team. Int J Gynaecol Obstet. 59 Suppl 2:S201-7.

Sabitu K, Alti-Mu'azu M, Musa AA, Ifenne DI, Essien ES, Golji NG, Adidu V, Mukaddas M. 1997
The effect of improving maternity services in a secondary facility, Zaria, Nigeria. The Zaria PMM Team. Int J Gynaecol Obstet. 59 Suppl 2:S99-106.

Shojania KG, Jennings A, Mayhew A, Ramsay CR, Eccles MP, Grimshaw J. The effects of on-screen, point of care computer reminders on processes and outcomes of care.. *Cochrane Database of Systematic Reviews* 2009, Issue 3. Art. No.: CD001096. DOI: 10.1002/14651858.CD001096.pub2

Thaddeus, S. & Maine, D. 1994, 'Too far to walk: maternal mortality in context', Social

Science & Medicine, vol. 38, no. 8, pp. 1091-1110.

UNICEF State of the World's Children, UNICEF New York 2009.

ANNEX 1: SEARCH STRATEGY AND TERMS
7/7/2010

To be used on MEDLINE search; and then adapted for EMBASE, CINAHL, CENTRAL and LILACS

1. exp transportation of patients/
2. time factors/
3. exp transportation/
4. health services accessibility/
5. telemedicine/
6. remote consultation/
7. patient transfer/
8. exp transportation/
9. communication/
10. information dissemination/
11. interdisciplinary communication/
12. bicycling/
13. motorcycles/
14. (bicycle or bicycles or bicycling or bike$ or cycle$).ti,ab.
15. transport$.ti,ab.
16. (car or cars or motor$ or vehicle$ or ambulance$ or donkey$).ti,ab.
17. (phone$ or telephone$ or cellphone$ or radio$).ti,ab.
18. telecommunications/
19. electronic mail/
20. radio/
21. Satellite Communications/
22. telefacsimile/
23. exp telephone/
24. exp Emergency Medical Service Communication Systems/
25. exp "referral and consultation"/
26. exp emergency service, hospital/
27. emergency medical services/
28. health services, indigenous/
29. emergency treatment/
30. triage/
31. first aid/
32. emergencies/
33. exp hospitalization/
34. delivery of health care/

35. after-hours care/

36. delivery of health care, integrated/

37. (pre-hospital or prehospital).ti,ab.

38. (responsive$ or referr$).ti,ab.

39. ((emergency or emergencies) adj10 (health or care or service$ or respons$)).ti,ab.

40. (emergency or emergencies).ti,ab.

41. delay$.ti,ab.

42. (timel$ or timing).ti,ab.

43. "antishock garment".ti,ab.

44. "anti-shock garment".ti,ab.

45. ((matern$ or deliver$) adj3 (wait$ or intermediate or outreach or out-reach or home$)).ti,ab.

46. ((birth$ or deliver$ or emergenc$) adj10 (plan$ or protocol$)).ti,ab.

47. ((first adj1 aid) or first-aid or (life adj1 saving)).ti,ab.

48. (emergenc$ adj2 obstetri$).ti,ab.

49. (obstetric adj5 care$).ti,ab.

50. (TBA or traditional birth attendant).ti,ab.

51. ((train$ or educat$) adj10 (matern$ or health$ or professional or midwive$ or midwife or nurs$)).ti,ab.

52. ((health or basic or comprehensive) adj10 (care or service$ or system$ or polic$)).ti,ab.

53. (BEmOC or EmOC or CEmOC).ti,ab.

54. (fund$ or financ$ or incentive$).ti,ab.

55. ((guideline$ or monitor$ or record$ or protocol$) adj10 (system$ or service$)).ti,ab.

56. ((health or community) adj5 (work$ or participant$ or profession$)).ti,ab.

57. (doctor$ or nurse$ or obstetr$ or midwife$ or midwive$ or attendant$).ti,ab.

58. or/1-57

59. maternal health services/ or maternal behavior/ or maternal-child nursing/ or maternal mortality/

60. pregnancy complications/ or pregnancy, high-risk/ or pregnancy complications, infectious/

61. delivery, obstetric/ or extraction, obstetrical/ or labor, obstetric/

62. pregnant women/

63. pregnancy/

64. (pregnancy or pregnant).ti,ab.

65. (antenatal or prenatal or antepartum or peripartum or postpartum).ti,ab.

66. (perinatal or postnatal).ti,ab.

67. (matern$ adj5 (mortality or morbidity)).ti,ab.

68. ((labour or labor) adj10 (deliver$ or birth$ or childbirth$)).ti,ab.

69. ((labour or labor) adj10 (infant$ or baby or babies or child$ or neonat$ or mother$ or matern$)).ti,ab.

70. (obstructed adj5 (labour or labor)).ti,ab.

71. (eclampsia or pre-eclampsia or ((genital or urin$) adj5 infect$)).ti,ab.

72. ((obstetric or postpartum or post-partum) adj5 (haemorrhag$ or hemorrhag$)).ti,ab.

73. (ruptur$ adj5 (uterine or uterus)).ti,ab.

74. or/59-73

75. 58 and 74

76. exp developing countries/

77. medically underserved area/

78. (developing adj5 countr$).ti,ab.

79. ((low income or low-income or middle income) adj5 (countr$ or area$ or population$ or city or cities or town$)).ti,ab.

80. exp africa/

81. exp central america/

82. exp latin america/

83. exp south america/

84. exp asia/

85. exp caribbean region/

86. exp caribbean community/

87. or/76-86

88. 58 and 74 and 87

89. exp randomized controlled trials/

90. randomized controlled trial.pt.

91. controlled clinical trial.pt.

92. exp random allocation/

93. (random$ or allocat$ or assign$).ti,ab.

94. exp clinical trials/

95. (clin$ adj25 trial$).ti,ab.

96. random$.ti,ab.

97. program evaluation/

98. exp epidemiologic studies/

99. exp epidemiologic research design/

100. epidemiologic methods/

101. exp empirical research/

102. feasibility studies/

103. pilot projects/

104. comparative study/

105. or/89-104

106. 88 and 105

107. human/

108. 106 and 107

109. (editorial or comment or letter or historical article).pt.

110. 108 not 109

111. case reports.pt.

112. 110 not 111

113. limit 112 to yr="1985 -Current"

114. limit 113 to yr="2006 -Current"

115. remove duplicates from 114

ANNEX 2 TIMELINE, PROJECT MANAGEMENT AND UPDATE

132 person days have been allocated to complete this work. Our timetable is as follows:

	Start date	End date
Registration of title with DFID	25th June 2010	1st July 2010
Preparation of protocol	1st June 2010	1st July 2010
DFID and External Review of protocol (if using peer review organized through 3ie, allow 3 weeks)	15th June 2010	15th July 2010
Study search	21st Jun 2010	14th Aug 2010
Assessment of study relevance	1st Aug 2010	30th Aug 2010
Extraction of data	1st Aug 2010	16th Sep 2010
Synthesis and/or statistical analysis	16th Sep 2010	15th Oct 2010
Preparation of draft report	1st Oct 2010	15th Nov 2010
DFID review of draft report (please allow 2 weeks)	15th Nov 2010	29th Nov 2010
Dissemination of draft report	1st Dec 2010	15th Jan 2010
Revision of draft report	1st Dec 2010	15th Jan 2010
External review of draft report (if using peer review organized through 3ie, allow 4 weeks for turnaround)	1st Dec 2010	15th Dec 2010
Revision and production of summaries	15th Dec 2010	28th Feb 2011

PROJECT MANAGEMENT: As the Principal Investigator, JH will be responsible for the deliverables of this research study. The University of Aberdeen will co-ordinate the administration of contracts for the members of the research team, all of whom will report to JH.

PLANS FOR UPDATING

This review is co-ordinated within Immpact at the University of Aberdeen, an international initiative for maternal mortality programme assessment, so we anticipate that we will be able to conduct a scoping of literature after two years to identify if a formal update is necessary and especially if a new intervention or observation of effectiveness is published. In general, updates of the literature search are recommended for Cochrane reviews after two years (Garritty et al 2010). If considerable new findings are anticipated, we would consider seeking resources to complete an update. It is unclear what methodologies can be used to update reviews in general (Moher et al 2008), although this may become clearer after the review is completed.

On-going studies identified will be described detailing the primary author, research questions(s), methods and outcome measures together with an estimate of the reporting date.
