# Supplementary material for: The Effectiveness of Emergency Obstetric Referral Interventions in Developing Country Settings: A Systematic Review
Source: PLoS Med. 2012 Jul 10;9(7):e1001264. doi: 10.1371/journal.pmed.1001264 (PMC3393680; doi:10.1371/journal.pmed.1001264)
Supplement: Text S3 — Search strategy. (DOC) [file pmed.1001264.s003.doc]

**The Effectiveness of Emergency Obstetric Referral Interventions in Developing Country Settings**

Search strategy for electronic databases

**(**MEDLINE search; adapted for other searches)

1. exp transportation of patients/

2. time factors/

3. exp transportation/

4. health services accessibility/

5. telemedicine/

6. remote consultation/

7. patient transfer/

8. exp transfer/

9. communication/

10. information dissemination/

11. interdisciplinary communication/

12. bicycling/

13. motorcycles/

14. (bicycle or bicycles or bicycling or bike$ or cycle$).ti,ab.

15. transport$.ti,ab.

16. (car or cars or motor$ or vehicle$ or ambulance$ or donkey$).ti,ab.

17. (phone$ or telephone$ or cellphone$ or radio$).ti,ab.

18. telecommunications/

19. electronic mail/

20. radio/

21. Satellite Communications/

22. telefacsimile/

23. exp telephone/

24. exp Emergency Medical Service Communication Systems/

25. exp "referral and consultation"/

26. exp emergency service, hospital/

27. emergency medical services/

28. health services, indigenous/

29. emergency treatment/

30. triage/

31. first aid/

32. emergencies/

33. exp hospitalization/

34. delivery of health care/

35. after-hours care/

36. delivery of health care, integrated/

37. "medical record"/ or "medical records"/

38. "health education"/

39. (pre-hospital or prehospital).ti,ab.

40. (responsive$ or referr$).ti,ab.

41. ((emergency or emergencies) adj10 (health or care or service$ or respons$)).ti,ab.

42. (emergency or emergencies).ti,ab.

43. delay$.ti,ab.

44. (timel$ or timing).ti,ab.

45. "antishock garment".ti,ab.

46. "anti-shock garment".ti,ab.

47. ((matern$ or deliver$) adj3 (wait$ or intermediate or outreach or out-reach or home$)).ti,ab.

48. ((birth$ or deliver$ or emergenc$) adj10 (plan$ or protocol$)).ti,ab.

49. ((first adj1 aid) or first-aid or (life adj1 saving)).ti,ab.

50. (emergenc$ adj2 obstetri$).ti,ab.

51. (obstetric adj5 care$).ti,ab.

52. (TBA or traditional birth attendant).ti,ab.

53. ((train$ or educat$) adj10 (matern$ or health$ or professional or midwive$ or midwife or nurs$)).ti,ab.

54. ((health or basic or comprehensive) adj10 (care or service$ or system$ or polic$)).ti,ab.

55. (BEmOC or EmOC or CEmOC).ti,ab.

56. (fund$ or financ$ or incentive$).ti,ab.

57. ((guideline$ or monitor$ or record$ or protocol$) adj10 (system$ or service$)).ti,ab.

58. ((health or communit$) adj5 (work$ or participant$ or profession$ or educat$ or fund$ or service$)).ti,ab.

59. (doctor$ or nurse$ or obstetr$ or midwife$ or midwive$ or attendant$).ti,ab.

60. or/1-59

61. maternal health services/ or maternal behavior/ or maternal-child nursing/ or maternal mortality/

62. pregnancy complications/ or pregnancy, high-risk/ or pregnancy complications, infectious/

63. delivery, obstetric/ or extraction, obstetrical/ or labor, obstetric/

64. obstetric care/

65. postpartum hemorrhage/

66. pre-eclampsia/

67. eclampsia/

68. labor complication/

69. pregnant women/

70. pregnancy/

71. (pregnancy or pregnant).ti,ab.

72. (antenatal or prenatal or antepartum or peripartum or postpartum).ti,ab.

73. (perinatal or postnatal).ti,ab.

74. (matern$ adj5 (mortality or morbidity)).ti,ab.

75. ((labour or labor) adj10 (deliver$ or birth$ or childbirth$)).ti,ab.

76. ((labour or labor) adj10 (infant$ or baby or babies or child$ or neonat$ or mother$ or matern$)).ti,ab.

77. (obstructed adj5 (labour or labor)).ti,ab.

78. (eclampsia or pre-eclampsia).ti,ab.

79. ((genital or urin$) adj5 infect$).ti,ab.

80. ((obstetric or postpartum or post-partum) adj5 (haemorrhag$ or hemorrhag$)).ti,ab.

81. (ruptur$ adj5 (uterine or uterus)).ti,ab.

82. or/61-81

83. 60 and 82

84. exp developing countries/

85. medically underserved area/

86. (developing adj5 countr$).ti,ab.

87. ((low income or low-income or middle income) adj5 (countr$ or area$ or population$ or city or cities or town$)).ti,ab.

88. exp africa/

89. exp central america/

90. exp latin america/

91. exp south america/

92. exp asia/

93. exp caribbean region/

94. exp caribbean community/

95. or/84-94

96. 60 and 82 and 95

97. exp randomized controlled trials/

98. randomized controlled trial.pt.

99. controlled clinical trial.pt.

100. exp random allocation/

101. (random$ or allocat$ or assign$).ti,ab.

102. exp clinical trials/

103. (clin$ adj25 trial$).ti,ab.

104. random$.ti,ab.

105. program evaluation/

106. program development/

107. exp epidemiologic studies/

108. exp epidemiologic research design/

109. "Case-Control Studies"/

110. "cohort studies"/

111. epidemiologic methods/

112. Cross-Sectional Studies/

113. exp empirical research/

114. feasibility studies/

115. pilot projects/

116. comparative study/

117. review.pt.

118. review.ti,ab.

119. (meta-analysis or meta-analyses).ti,ab.

120. meta-analysis.pt.

121. "Costs and Cost Analysis"/

122. health care financing/

123. cost$.ti,ab.

124. or/97-123

125. 96 and 124

126. human/

127. 125 and 126

128. (editorial or comment or letter or historical article).pt.

129. 127 not 128

130. case reports.pt.

131. 129 not 130

132. limit 131 to yr="1985 -Current"

Note: We originally intended to include interventions used to stabilise and/or treat the woman before arrival at the facility/during referral, including obstetric first-aid techniques, use of the anti-shock garment and drugs for community/lower-level health facility use such as oxytocin and misoprostol. On further consideration, we felt that these interventions were not designed to reduce Phase II delays, but aimed to either obviate the need for referral, or to keep a woman alive while awaiting referral, so the articles retrieved were excluded from the study.
